# The Associations of Objectively Measured Physical Activity and Sedentary Time with Cognitive Functions in School-Aged Children

**DOI:** 10.1371/journal.pone.0103559

**Published:** 2014-07-25

**Authors:** Heidi J. Syväoja, Tuija H. Tammelin, Timo Ahonen, Anna Kankaanpää, Marko T. Kantomaa

**Affiliations:** 1 LIKES – Research Center for Sport and Health Sciences, Jyväskylä, Finland; 2 Department of Psychology, University of Jyväskylä, Jyväskylä, Finland; 3 Department of Epidemiology and Biostatistics, MRC–HPA Centre for Environment and Health, Imperial College London, London, United Kingdom; Tokyo Metropolitan Institute of Medical Science, Japan

## Abstract

Low levels of physical activity among children have raised concerns over the effects of a physically inactive lifestyle, not only on physical health but also on cognitive prerequisites of learning. This study examined how objectively measured and self-reported physical activity and sedentary behavior are associated with cognitive functions in school-aged children. The study population consisted of 224 children from five schools in the Jyväskylä school district in Finland (mean age 12.2 years; 56% girls), who participated in the study in the spring of 2011. Physical activity and sedentary time were measured objectively for seven consecutive days using the ActiGraph GT1M/GT3X accelerometer. Self-reported moderate to vigorous physical activity (MVPA) and screen time were evaluated with the questions used in the “WHO Health Behavior in School-aged Children” study. Cognitive functions including visual memory, executive functions and attention were evaluated with a computerized Cambridge Neuropsychological Test Automated Battery by using five different tests. Structural equation modeling was applied to examine how objectively measured and self-reported MVPA and sedentary behavior were associated with cognitive functions. High levels of objectively measured MVPA were associated with good performance in the reaction time test. High levels of objectively measured sedentary time were associated with good performance in the sustained attention test. Objectively measured MVPA and sedentary time were not associated with other measures of cognitive functions. High amount of self-reported computer/video game play was associated with weaker performance in working memory test, whereas high amount of computer use was associated with weaker performance in test measuring shifting and flexibility of attention. Self-reported physical activity and total screen time were not associated with any measures of cognitive functions. The results of the present study propose that physical activity may benefit attentional processes. However, excessive video game play and computer use may have unfavorable influence on cognitive functions.

## Introduction

In past decades, our lifestyles have become increasingly inactive [1]; only one-third of children are sufficiently active according to current physical activity recommendations [2]. Low levels of physical activity have raised concerns over the effects of a physically inactive lifestyle on children’s physical health and, recently, also on children’s learning, especially on cognitive prerequisites of learning [3].

Previous studies have shown that physical activity enhances neurocognitive function and protects against neurodegenerative diseases in elderly [4]–[6]. During past few years, physical activity has been linked to enhanced cognition also in children. The meta-analytic study of Sibley & Etnier [7], showed the significant overall positive association between physical activity and cognition in children. In addition, in the study of Ruiz et al. [8] leisure time physical activity was associated with better cognitive performance in adolescents. Moreover, Ardoy et al. [9] reported that children participating in high intensity physical education intervention improved their cognitive performance compared to control children.

Regular physical activity has been especially linked to executive functions [10]. Executive functions (also called executive control/cognitive control) are the collection of higher-order cognitive processes controlling goal-directed actions. Core executive functions are inhibitory control (including selective attention and the inhibition of inappropriate or interfering responses), working memory and mental flexibility [11]. School-based interventions has shown that increased physical activity may improve children’s inhibitory control [12], planning ability [13] and working memory performance [14], [15]. In addition, in recent studies, children with high aerobic fitness have demonstrated better inhibitory control [16]–[18] as well as better working memory performance [19] than less-fit children.

However, evidence of the favorable effects of physical activity on cognitive functions in healthy children and adolescents is still somewhat inconsistent [20]–[22] and based on scarce research data [10]. Significantly, physical fitness has often been used as a proxy indicator of regular physical activity, without direct measurement of physical activity levels. This is particularly important, because in childhood, habitual physical activity is rarely intensive and lengthy enough to enhance aerobic fitness, and therefore, the relationship between physical activity and fitness may not be meaningful [23]. Moreover, few studies have measured a broad range of cognitive functions, highlighting the need for new studies to clarify the benefits of physical activity on different dimensions of cognitive functions.

Besides physical activity, sedentary behavior and excessive media use may be associated with cognitive function in children and youth. Extensive screen time has been linked to elevated risk of attention and learning difficulties [24]–[27] and decreased verbal memory performance [28]. However, in other recent studies, screen time had no association with visuospatial cognition [29], and even had a positive association with enhanced attentional skills [30] and higher-developed language skills [31] in children and adolescents. Diverging research results indicates that the association of sedentary behavior and cognition is more complicated than previously believed and needs clarification.

To our knowledge, no previous studies have examined the associations of objectively measured overall physical activity and sedentary time on cognitive functions in children. However, as the rates of childhood physical inactivity are increasing worldwide, it is important to better understand the potential effects of lack of physical activity and excessive sedentary time on cognitive prerequisites of learning. The purpose of this study was to examine how objectively measured and self-reported physical activity are associated with cognitive functions in school-aged children. In addition, this study aimed to determine how objectively measured sedentary time and self-reported screen time are associated with children’s cognitive functions. We hypothesized that physical activity is positively, and both sedentary time and screen time are inversely, associated with cognitive functions.

## Method

### Ethics Statement

The study was approved by the Ethics Committee of the University of Jyväskylä, and followed the principles of the Declaration of Helsinki and the Finnish legislation. Participation in the study was voluntary, and all participants had the right to drop out of the study at any time without a specific reason. Only children with a fully completed consent form (Certificate of Consent signed by a parent/guardian and the child) on the day of the first measurements were included in the study.

### Participants

During spring 2011, 475 fifth and sixth graders were invited to participate in the study. 277 children (participation rate 58%) from five schools in the Jyväskylä school district in Central Finland participated in the study. 230 of the 277 children were selected to participate in cognitive tests according to successful objective measurement of physical activity. If a child’s measurement did not succeed because of technical problems or the child did not remember to wear the accelerometer, they were not invited to the cognitive tests. Seven children (3 boys, 4 girls) were excluded from analysis because, according to their parents’ survey, they had physical disabilities, chronic diseases or severe learning disabilities. The final sample size used in the analyses was 224.

### Cognitive functions

Cognitive functions (Table 1) were assessed using the Neuropsychological Test Automated Battery (CANTAB) (a PaceBlade Slimbook P110 tablet PC with a 12-inch touch-screen monitor and Windows XP Professional operating system, CANTABeclipse version 3). The test battery was run individually in a silent location with the guidance of trained research assistants and in accordance with the standard instructions. The execution required about 45 minutes for each individual.

**Table 1:** Summary of the CANTAB tests used to measure different dimensions of cognitive function.

Dimension of cognitive function	Test	Abbreviation
Visual memory	1. Pattern Recognition Memory	PRM
Executive function	2. Spatial Span	SSP
	3. Intra-Extra Dimensional Set Shift	IED
Attention	4. Reaction Time	RTI
	5. Rapid Visual Information Processing	RVP

Visual memory was assessed with a Pattern Recognition Memory (PRM) test. PRM measures recognition memory of visual patterns in a two-choice forced discrimination paradigm. In this test, children had to remember the presented geometric patterns and discriminate them from the novel patterns. The score of the task is the number of correct responses.

Executive functions were assessed with Spatial Span (SSP) and Intra-Extra Dimensional Set Shift (IED) tests. SSP measures the length of the visuospatial working memory span based on the Corsi blocks task [32]. In this test, specified number of white boxes changed their color one by one and children had to reproduce the same sequence by touching the boxes in the same order the boxes changed their color. The score of the task is the maximum number of items that the child can successfully remember in the correct order. IED is based on the Wisconsin Card Sorting test [33] and measures sifting and flexibility of attention. Specifically, it measures the ability to maintain attention to different stimuli within a relevant dimension, and shift attention to a previously irrelevant dimension. There are nine stages with increasing difficulty in this task. The children were instructed to choose one of the two different dimensions: one is correct, and the other is incorrect. According to immediate feedback, they were expected to choose the correct pattern and learn the rule. The score in this task is based on the number of stages completed.

The tests assessing attention were Reaction Time (RTI) and Rapid Visual Information Processing (RVP). RTI measures children’s reaction time and speed of response to a visual target. In the unpredictable five-choice condition, a yellow spot appeared randomly in one of the five circles on the screen. Children were instructed to hold down the press pad button until they saw the yellow spot and then touch the middle of the correct circle as quickly as possible. The total score of this task is the sum of reaction time (ms) and movement time (ms). RVP is similar to the Continuous Performance Task measuring sustained attention. In this test, children had to touch the press pad button every time they discriminate the target sequence (digits 3, 5, and 7) from the digits appearing in a pseudo-random order at the rate of 100 digits per minute. The score of this task is RVP A’, which measures the child’s skill at detecting target sequences (3, 5, 7) from a pseudo-random sequence of numbers (range 0.00 to 1.00; bad to good).

Internal reliability, assessed with Cronbach’s alpha reliability coefficients, was 0.49 for the RVP A’, 0.65 for the PRM number of correct responses, 0.66 for the RTI five-choice reaction time and 0.87 for the RTI five-choice movement time. For hampering tests (SSP and IED), Cronbach’s alpha could not be determined.

### Objectively measured physical activity and sedentary time

The ActiGraph GT1M/GT3X accelerometers with vertical axel were used to measure children’s moderate to vigorous physical activity (MVPA) and sedentary time. The accelerometer was worn on the right hip with an elastic waistband during waking hours for seven consecutive days. During bathing, swimming, and other water activities, the monitor was requested to be removed because it was not water-resistant. The ActiLife accelerometer software (ActiLife version 5; http://support.theactigraph.com/dl/ActiLife-software) was used to collect the data. Epoch length was 10 seconds and non-wearing time 30 minutes. Customized software was used for data reduction and analysis. A cut-off value of 2,296 counts per minute was used for MVPA [34], and 100 counts per minute for sedentary time. Children were included in the analysis if they had valid data for at least 500 minutes per day on two weekdays and on one weekend day. In order to compare children, who had worn the accelerometers for different amounts of time per day, objectively measured sedentary time was expressed as percentage of daily registration time.

### Self-reported physical activity and screen time

Physical activity and screen time were assessed with a self-reported questionnaire used earlier in the WHO Health Behavior in School-aged Children (HBSC) study [35]. Self-reported MVPA was measured with the following question: “Over the past 7 days, on how many days were you physically active for a total of at least 60 minutes per day?” The response categories were as follows: 0 days, 1 day, 2 days, … 7 days. There was a short description about what kind of physical activity should be taken into account when answering the question: “In the next question, physical activity is defined as any activity that increases your heart rate and makes you get out of breath some of the time.” Examples included running, walking quickly, rollerblading, biking, dancing, skateboarding, swimming, snowboarding, cross-country skiing, soccer, basketball, and Finnish baseball. Test-retest agreement for self-reported MVPA has been very good (ICC = 0.82) [36], [37]. Self-reported screen time was evaluated with the question: “About how many hours a day do you usually a) watch television (including videos), b) play computer or video games, or c) use a computer (for purposes other than playing games, for example, emailing, chatting, or surfing the Internet or doing homework) in your free time?” The response options were as follows: not at all, about half an hour per day, about an hour a day, about two hours per day, … about five hours per day or more. Children responded separately for both weekdays and weekends. Test-retest agreement for watching television (ICC = 0.72–0.74) and for playing computer or video games (ICC = 0.54–0.69) has been substantial, and fair to moderate (ICC = 0.33–0.50) for using the computer [37]. Total daily screen time averages were calculated by adding these three questions, including weekdays and weekends, together.

### Potential confounders

The parent or the child’s main caregiver filled in a questionnaire including the mother and father’s education, family income, marital status, and children’s learning difficulties and need for remedial education. The highest level of parental education, which was calculated from the mother’s and father’s education, was categorized as tertiary level education (1) and basic or upper secondary education (0). Marital status of the main caregiver was categorized as married or cohabiting (1) and divorced or single/widow (0). Children’s learning difficulties and need for remedial education were categorizes as yes (1) and no or don’t know (0).

### Statistical analysis

The SPSS 19.0 for Windows statistical package (SPSS (2010) IBM SPSS Statistics 19 Core System User’s Guide (SPSS Inc., Chicago, IL)) and the Mplus statistical package (Version 7; Muthèn & Muthèn, 1998–2012) [38] were used for the statistical analyses. Pearson’s correlation coefficients were calculated to estimate preliminary associations between objectively and subjectively measured physical activity, sedentary behavior, cognitive tests and potential confounders. Structural equation modeling was applied to examine physical activity and sedentary time in association with cognitive function. Structural equation modeling was used because it enables to estimate the measurement errors away and, therefore, increases the reliability of cognitive tests. Single scores of every problem or level of the tests were applied instead of the total score. Item parcels [39] were constructed to combine the large number of these single scores for each latent factor. These parcels were then used as indicators for latent variables. Multiple logistic regression was used to examine physical activity and sedentary time in association with cognitive function, when the outcome was dichotomous. Full information maximum likelihood (FIML) estimation with robust standard errors (MLR) was used under the assumption of data missing at random. Gender, the highest level of parental education and child’s need for remedial education were chosen to represent different aspects of potential confounders and were added to the main analysis. In order to avoid multicollinearity, highly correlated objectively measured MVPA and sedentary time were added to the model using a Cholesky factoring of the predictors [40]. The Satorra–Bentler scaled χ2-test, the comparative fit index (CFI), the Tucker–Lewis Index (TLI), the root mean square error of approximation (RMSEA) and the standardized root-mean-square residual (SRMR) were used to evaluate the goodness-of-fit of the models. The model fits the data well if the p-value for the χ^2^-test is non-significant, CFI and TLI values are close to 0.95, the RMSEA value is below 0.06 and the SRMR value is below 0.08 [41].

## Results

The mean age of the children was 12.2 years and 56% of the children were girls (Table 2). 71% of children’s mothers and 56% of children’s fathers had tertiary level education, and 76% of parents were married or cohabiting. 5% of children had a diagnosed learning difficulty and 13% of children needed remedial education, according to their parent’s reports.

**Table 2:** Sample characteristics according to gender and overall participants.

	Boys			Girls			All			p^a^
	Mean	SD	N	Mean	SD	N	Mean	SD	N	
Age (years)	12.2	0.6	97	12.2	0.6	127	12.2	0.6	224	0.984
**Measurements of cognitive function**										
PRM no. of correct	20.9	2.8	97	20.9	2.2	127	20.9	2.5	224	0.981
SSP span length (scale 0–9)	6.6	1.3	97	6.7	1.4	127	6.6	1.3	224	0.561
RTI five-choice (ms)	623	78	97	680	98	127	655	94	224	<0.001
RVP A’ (scale 0.00–1.00)^b^	0.97	0.02	97	0.97	0.03	127	0.97	0.02	224	0.946
IED no. of children who completed the test (%)	67		97	66		127	67		224	0.891
**Measurements of physical activity and sedentary behavior**										
Objectively measured MVPA (min/day)^c^	60.3	22.4	89	56.9	16.9	118	58.4	19.5	207	0.231
Objectively measured sedentary time (%/day)^d^	66.1	5.9	89	67.9	5.1	127	67.2	5.5	207	0.013
Self-reported MVPA (d/week with ≥60 min MVPA)	5.0	1.8	95	5.0	1.5	127	5.0	1.6	222	0.923
Self-reported total screen time (h/day)	4.0	2.0	95	3.4	1.8	127	3.7	1.9	222	0.015
TV	1.6	1.0	96	1.6	0.9	127	1.6	0.9	223	0.982
Computer/video games	1.4	0.9	95	0.7	0.8	127	1.0	0.9	222	<0.001
Computer use (other than playing)	1.0	0.8	96	1.1	0.8	127	1.0	0.8	223	0.244

Abbreviations: SD, standard deviation; MVPA, moderate to vigorous physical activity; PRM, Pattern Recognition Memory; SSP, Spatial Span; RTI, Reaction Time; RVP, Rapid Visual Information Processing; IED, Intra-Extra Dimensional Set Shift.

a P-values for the gender differences (T-test).

b A’ indicates the result in RVP test. The scale is 0.00–1.00, whereas 0.00 indicates a poor result and 1.00 a good result.

c MVPA measured with the ActiGraph accelerometer using a cut-off value of 2,296 counts per minute.

d Sedentary time measured by the ActiGraph accelerometer using a cut-off value 100 counts per minute and expressed as percentage of daily monitoring time (%/day).

Based on objective physical activity measurements, children had, on average, 58 minutes of MVPA per day, with no statistically significant gender difference (Table 2). However, girls spent more of their waking hours sedentary than boys (Table 2). Based on self-reports, children reported at least 60 minutes of MVPA a day for 5 days per week on average, with no significant difference between boys and girls (Table 2). Boys reported more total screen time than girls, especially they spent more time playing computer or videogames than girls (Table 2). In cognitive tests, boys were faster than girls in the RTI test, but no significant gender differences were observed in performance in the PRM, SSP, RVP or IED tests.

**Figure 1:**
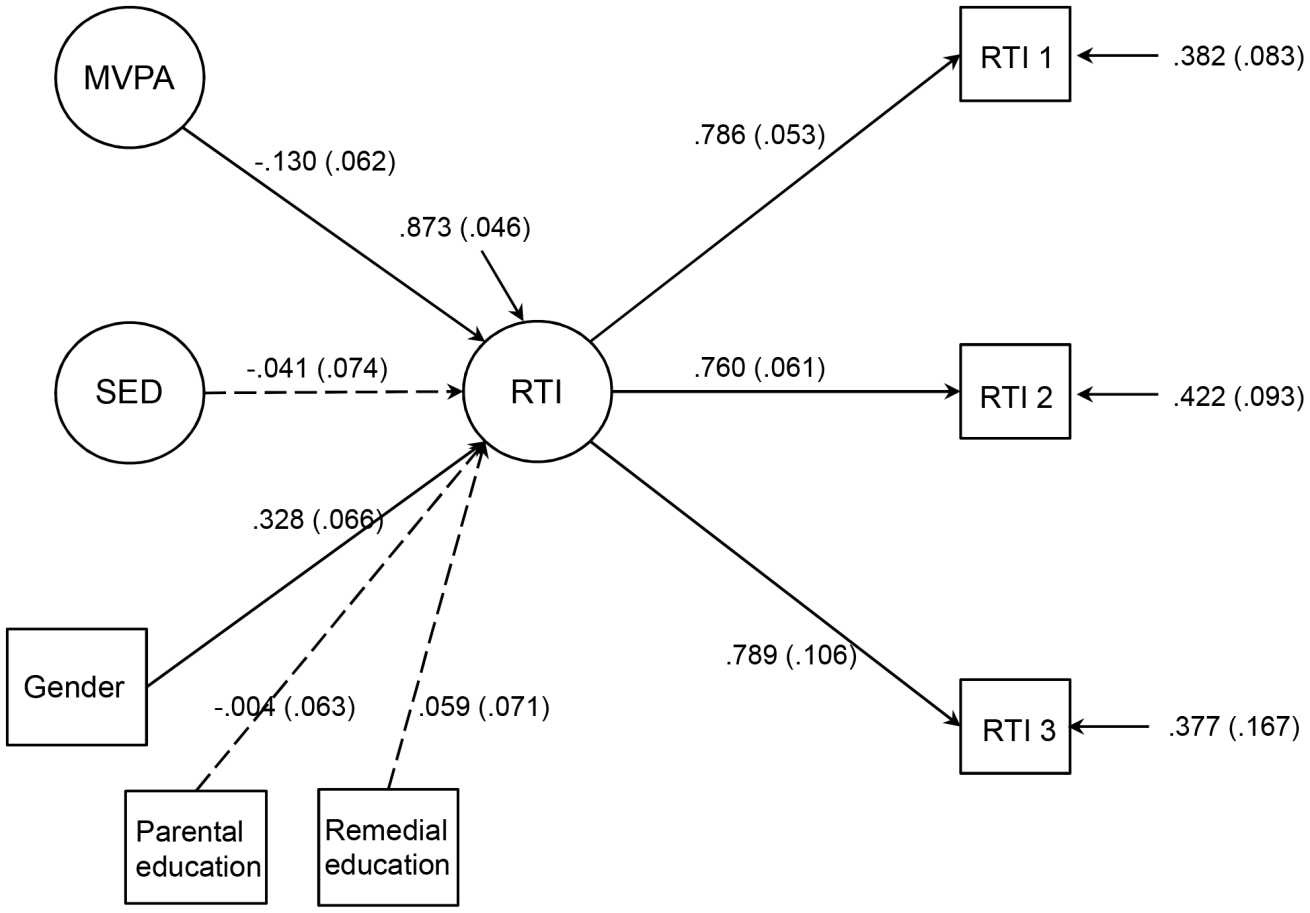
Objectively measured physical activity and performance in attentional reaction time test. This figure presents the estimation results of the model for the associations of objectively measured moderate to vigorous physical activity (MVPA), sedentary time (SED) and the Reaction Time (RTI) test. Standardized parameter estimates and standard errors are presented. For structural equation modeling, three subscales of the RTI (RTI 1, RTI 2, RTI 3) (divided by 10) were formed from 15 patterns of RTI. The RTI test result is in milliseconds, where faster time indicates better performance. Confounding factors, gender (female), the highest level of parental education (tertiary level) and child’s need for remedial education (yes) were taken into account. Highly correlated objectively measured MVPA and sedentary time were added to the model as latent variables.

For structural equation modeling, three subscales of RTI were formed from 15 individual patterns of the RTI, and each subscale divided by 10. Each subscale loaded on the hypothesized factor. The model for the associations of objectively measured MVPA, sedentary time (SED) and performance in the Reaction Time (RTI) test is presented in Figure 1. The goodness-of-fit statistics of the model were good (χ^2^ (10) = 14.52 p = 0.763, CFI = 0.979, TLI = 0.948, RMSEA = 0.045, SRMR = 0.018). Objectively measured MVPA was negatively associated with the RTI five-choice test score (ms), whereas objectively measured sedentary time was not associated with the RTI five-choice test score after adjusting for gender, the highest level of parental education and child’s need for remedial education (Table 3).

**Table 3:** The associations between children’s cognitive processes and objectively measured physical activity, sedentary time and self-reported screen time.

	B	SE	95% CI	P^g^
**Reaction Time (RTI)** a				
Objectively measured MVPA^b^	−0.130	0.062	−0.253, −0.008	0.037
Objectively measured sedentary time^c^	−0.041	0.074	−0.186, 0.104	0.581
**Rapid Visual Information Processing (RVP)** d				
Objectively measured MVPA	−0.040	0.093	−0.223, 0.143	0.669
Objectively measured sedentary time	0.305	0.078	0.153, 0.457	0.000
**Spatial Span (SSP)** e				
Self-reported viewing of TV^f^	−0.003	0.067	−0.134, 0.129	0.970
Self-reported playing of computer/video game^f^	−0.179	0.079	−0.333, −0.024	0.023
Self-reported use of computer (other than playing)^f^	0.094	0.068	−0.040, 0.227	0.171

Abbreviations: B, estimate; SE, standard error; CI, confidence interval; p, P-value; MVPA, moderate to vigorous physical activity.

a RTI measures children’s reaction time and speed of response to a visual target in milliseconds, where faster time indicates better performance.

b MVPA measured with the ActiGraph accelerometer using a cut-off value of 2,296 counts per minute and expressed as min/day.

c Sedentary time measured by the ActiGraph accelerometer using a cut-off value of 100 counts per minute and expressed as percentage of daily monitoring time (%/day).

d RVP measures the sustained attention. The score of this task is RVP A’, where range is 0.00 to 1.00; bad to good.

e SSP measures length of the visuospatial working memory span. The score of this task is the maximum number of items that the child can successfully reproduce.

f Self-reported viewing of television, playing of computer/video games and use of computer (other than playing) are expresses as h/day.

g P-values for parameter estimates.

Models have been adjusted with gender (female), the highest level of parental education (tertiary level) and child’s need for remedial education.

**Figure 2:**
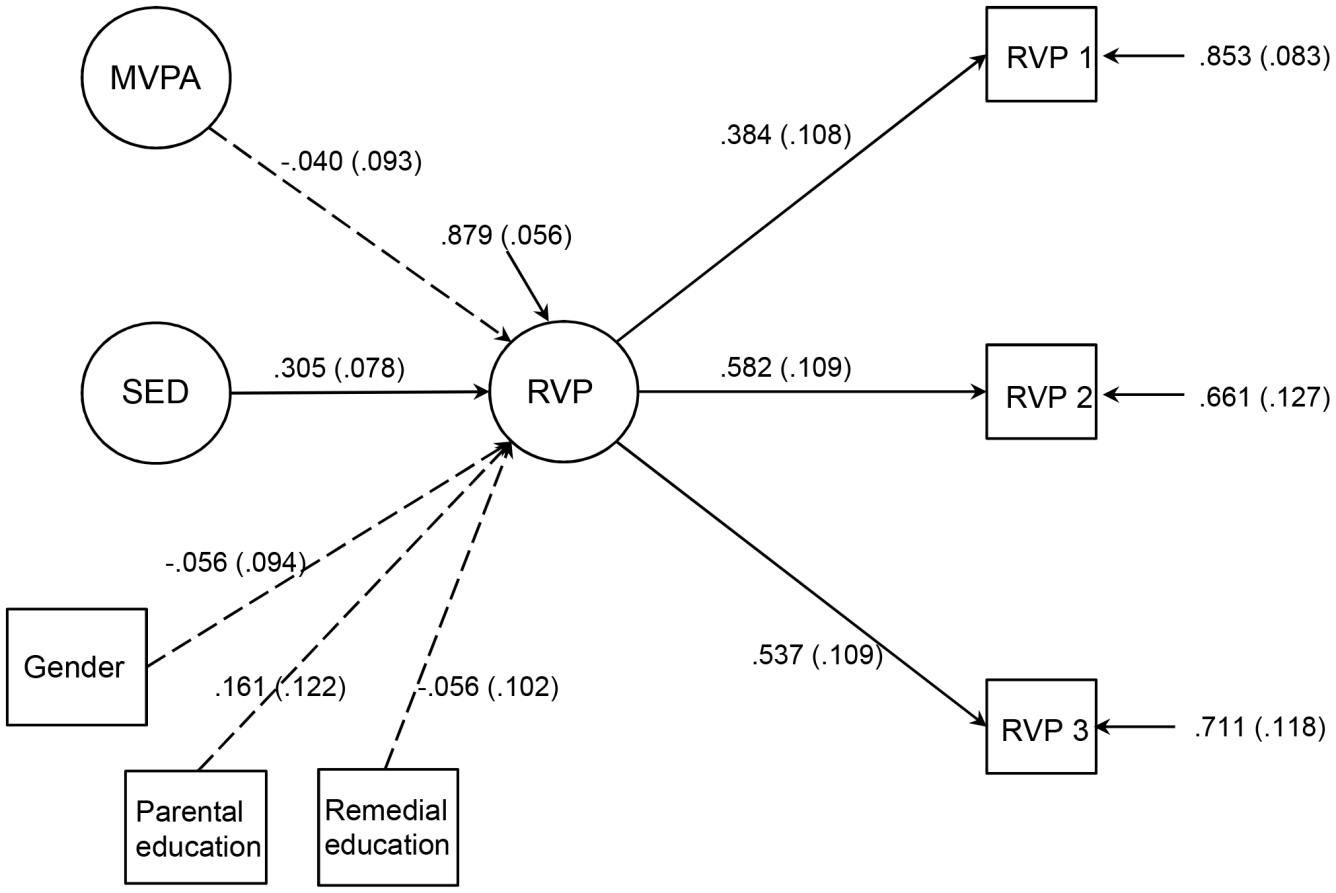
Objectively measured sedentary time and performance in sustained attention test. This figure presents the estimation results of the model for the associations of objectively measured MVPA, sedentary time (SED) and the Rapid Visual Information Processing (RVP) test. Standardized parameter estimates and standard errors are presented. The RVP (multiplied by 10) of the three blocks of the test (RVP 1, RVP 2, RVP 3) were used as indicator variables in the structural equation modeling. The scale for the RVP test result (A’) is 0.00–1.00, where 0.00 indicates a poor result and 1.00 a good result. Confounding factors, gender (female), the highest level of parental education (tertiary level) and child’s need for remedial education (yes) were taken into account. Highly correlated objectively measured MVPA and sedentary time were added to the model as latent variables.

The RVP A’ (multiplied by 10) scores of the three blocks of the test were used as indicator variables in the structural equation modeling. Each block loaded on the hypothesized factor. The model for the associations of objectively measured MVPA, sedentary time (SED) and performance in the Rapid Visual Information Processing (RVP) test is presented in Figure 2. The goodness-of-fit statistics of the model were good (χ^2^ (10) = 9.45 p = 0.490, CFI = 1.000, TLI = 1.010, RMSEA = 0.000, SRMR = 0.028). Objectively measured sedentary time was positively associated with RVPA’, whereas objectively measured MVPA was not associated with RVPA’ after adjusting for gender, the highest level of parental education and child’s need for remedial education (Table 3).

Self-reported playing of computer/video games was negatively associated with SSP span length after adjusting for gender, the highest level of parental education and child’s need for remedial education (Table 3). The model was fully saturated. Self-reported use of computer for other purposes than playing was negatively associated with IED number of children who completed the test (Table 4).

**Table 4:** The associations between children’s working memory capacity and self-reported screen time.

	OR	95% CI
**Intra-Extra Dimensional Set Shift (IED)** a		
Self-reported viewing of TV^b^	0.868	0.623, 1.210
Self-reported playing of computer/video game^b^	1.321	0.943, 1.850
Self-reported use of computer (other than playing)^b^	0.639	0.421, 0.972

Abbreviations: OR, odds ratio; CI, confidence interval.

a IED measures sifting and flexibility of attention and was categorized as 1 children who completed the test and 0 children who did not completed the test.

b Self-reported viewing of television, playing of computer/video games and use of computer (other than playing) are expresses as h/day.

Model has been adjusted with gender (female), the highest level of parental education (tertiary level) and child’s need for remedial education.

## Discussion

According to the results of this study, a high level of objectively measured MVPA was associated with good performance in the reaction time test (RTI), which measures children’s reaction time and the speed of response to a visual target. In addition, a high level of objectively measured sedentary time was associated with good performance in the sustained attention test (RVP). However, objectively measured physical activity or sedentary time were not associated with any other assessments of cognitive functions. High amount of self-reported playing of computer or video games was associated with weaker performance in Spatial Span (SSP) test measuring visuospatial working memory. Moreover, self-reported use of computer for other purposes than playing was negatively associated with performance in Intra-Extra Dimensional Set Shift (IED) test, which measures sifting and flexibility of attention. Self-reported physical activity, total screen time or television viewing had no association with any of the cognitive tests measuring visual memory, executive functions or attention.

### Physical activity and cognitive functions

In our study, a high level of objectively measured physical activity was associated with better performance in attentional reaction time test. Recent study of Spitzer and Hollmann [42] supports our finding by reporting that implementation of physical activity had positive effects on children’s attention. In addition, recent intervention study of Chaddock-Heyman et al. [12] showed that physical activity enhances children’s performance in inhibitory control task requiring selective attention and inhibition of interfering responses (e.g. the Eriksen flanker task; [43]). Similarly, according to Castelli et al. [44] engagement in vigorous physical activity was positively associated with performance in inhibitory control task. However, in the studies of Fisher et al. [14], Davis et al. [13] and Puder et al. [20] physical activity intervention had no effect on children’s attentional processes. Previous studies have also reported that physically fit children outperform their less fit peers inhibitory control task [17], [18], [45], but also no differences in inhibitory control performance between physically fit and less fit children [22], [44].

Physical activity may enhance attentional processes and other cognitive functions through different mechanisms. It has been suggested that physical activity may improve brain volume in regions supporting executive functions [19], [46]; produce specific changes in the activity patterns in the brains [12], [13]; increase the levels of brain-derived neurotrophic factor (BDNF) [47], [48] and enhance cerebrovascular function [49]; all of which may mediate the effects of physical activity on cognition. In addition, motor function has shown to be closely connected to children’s cognitive and academic skills and development [50], [51], and may be an important factor driving the effects of physical inactivity on cognitive prerequisites of learning [52]. Furthermore, obesity has been related to poorer academic [52] and cognitive performance [53] and may, thereby, be one factor mediating the association of physical activity and cognition. Moreover, participation in physical activities is often a social phenomenon offering opportunities for interaction with other children and adults, and this interaction may also have a significant impact on children’s cognitive development and learning. However, this has rarely been taken into account in research [54]. Finally, physical activity may facilitate cognitive function through the cognitive demands inherent in the structure of goal-directed and engaging exercise [55].

In the present study, neither objectively measured nor self-reported physical activity was associated with visual memory, working memory, sifting and flexibility of attention or sustained attention performance. These results are in line with studies showing that physical activity is not necessary associated with all domains of cognitive functions [13]–[15], [20], [21]. These diverging results indicate the importance of future studies to define these associations. Some of the cognitive tests used in the present study were not able to differentiate healthy 12-year-old children. Neuropsychological test batteries have originally been developed to detect neurocognitive deficits, and due to that, the tests may be too easy for healthy children. In the present study, particularly in the tests of visual memory (PRM), sifting and flexibility of attention (IED) and sustained attention (RVP), children, on average, achieved very high results, which may partly explain the lack of association between physical activity and cognitive test results. Additionally, Stroth et al. [22] speculated that one reason behind the result of aerobic fitness not being associated with children’s performance in cognitive control tasks may be the ceiling effect.

In previous studies, aerobic fitness has often been used as a proxy measure of regular physical activity, which might contribute to diverging results: physical activity is behavior, which increases energy expenditure and occurs within a cultural context, while physical fitness is an adaptive state of the human body, affected by heritable and environmental factors and physical activity. Especially in childhood, both the levels of physical activity and physical fitness may vary independently of each other due to growth, maturation and aging [23], [56]. In addition, aerobic fitness measures are often confounded by adiposity and obesity in childhood [57], which are also potential factors attenuating cognitive and academic performance [53]. Moreover, the definitions, patterns and measurements of cognitive functions and physical activity have varied across different studies. Therefore, it is difficult to directly compare and summarize the results of earlier studies and determine the specific benefits regular physical activity may have on cognitive functions.

Our finding that only objectively measured physical activity was associated with attentional reaction time may reflect the difference between objective and subjective measurements of physical activity. Accelerometer-measured MVPA mainly illustrates cardiovascular activity with increased heart rate and respiratory frequency, while self-reported physical activity may represent different constructs and contexts. Self-reported physical activity may include skill-specific types of physical activities, which require balance and agility but hardly accumulate activity counts [58]. Our results may indicate that moderate to vigorous intensity exercise that increases cardiovascular function has benefits on attentional processes.

### Sedentary behavior and cognitive functions

Previous studies have largely suggested that screen-based sedentary behaviors have an unfavorable effect on children’s cognition, especially on attention and learning difficulties [24]–[27]. The results of the present study supports the previous results by showing a negative association between self-reported computer/video game playing and visuospatial working memory as well as negative association between self-reported use computer and shifting and flexibility of attention. Drowak et al. [28], reported also declines in verbal memory performance after computer game exposure. However, some previous studies have reported that screen-based sedentary behavior, especially videogames, is linked to enhanced cognitive skills [59]–[61]. According to Dye and Matthew [30], children who used to play video games had faster reaction times in attention control tests (ANT) without a notable loss in accuracy compared to non-players, indicating that action game players made faster correct responses to targets and had more resources to process distractions. Bittman et al. [31] reported that computer use was associated with higher-developed language skills.

In the present study, some children spent excessive amounts of time in front of the screens on their free time: one fifth of the children reported having screen time about 5 or more hours per day. Excessive amounts of screen time may displace activities involving learning opportunities and increase children’s impulsive behavior, and eventually decrease academic skills [62].

Disadvantages, but also benefits of screen-based sedentary behavior on cognitive functions may be explained by the content of the screen time: not all screen time has an equal role in benefitting or impairing children’s cognitive skills and learning [63], [64]. For example, Ennemoser & Schneider [65] reported that educational program viewing was positively, but entertainment program viewing negatively, correlated with reading speed and comprehension in children. In the study of Feng et al. [66], action game training in young adults improved performance and attenuated the gender differences favoring males in spatial attention test, while control subjects who played a non-action game showed no improvements. According to Kuhn et al. [67], video game playing may induce structural brain plasticity in the areas important to spatial navigation, strategic planning, working memory and motor performance. On the other hand, in the study of Drowak et al. [28], interactive computer game play resulted significant declines in verbal memory performance and slow wave sleep, which is important for memory consolidation, whereas viewing exciting films had no effects. Finally, it should be kept in mind that computer-based cognitive assessments may require similar cognitive skills as video and computer games, which would favor children who play a lot of video and computer games.

Most of the previous studies have used self-reported methods assessing sedentary behavior. In the present study, objectively measured sedentary time was positively associated with sustained attention; children who spent more time being sedentary achieved higher scores in sustained attention test. Objectively measured sedentary time is a summary measure of all kinds of sedentary behaviors, including various activities such as screen time, reading, doing homework, interaction with friends, et cetera. During some sedentary activities, such as reading and doing homework, sustained attention and distraction exclusion are needed, which may explain objectively measured sedentary time being associated with good performance in sustained attention test.

In the present study, however, objectively measured sedentary time was not associated with visual memory, working memory, set-sifting/mental flexibility or attentional reaction time. In addition, total screen time or television viewing were not associated with cognition. Neither did Ferguson et al. [29] observe any association between video game playing and visuospatial cognition. Moreover, in two recent studies [31], [68], television viewing was not associated with cognitive and language skills after adjusting for the parent’s role in monitoring and involvement in the child’s media use.

### Strength and limitations of the study

To our knowledge, this was the first study examining the association of objectively measured overall physical activity and sedentary time with cognitive functions in children. Conclusions regarding causality of the observed associations cannot be drawn due to the cross-sectional design. In addition, the content of sedentary time and screen-based sedentary behavior was not assessed, which limits the interpretation of the results concerning sedentary behavior. Moreover, some cognitive tests used in the present study may have been too easy for 12-year-old healthy children, and did not optimally discriminate children’s performance. Furthermore, pubertal timing, motor skills and fitness were not assessed, which limits the interpretation of the results.

### Future direction

Studies with longitudinal designs or randomized controlled trials are needed to clarify the effects of total physical activity and sedentary behavior on cognitive prerequisites of learning. Future studies should assess what kind of physical activity or sedentary behavior affects specific kinds of cognition, as well as the mechanisms behind these associations. In the future, social interaction and context-related factors should be considered to be taken into account. In addition, the cognitive tests should be chosen so as to measure a wide range of cognitive performance.

### Conclusion

In this study, objectively measured physical activity and sedentary time were positively associated with attentional processes, but not with other domains of cognitive functions. Self-reported computer/video game play was negatively associated with visuospatial working memory, whereas computer use was negatively associated with shifting and flexibility of attention. Self-reported physical activity and total screen time were not associated with any of the cognitive tests measuring visual memory, executive functions or attention in children. The results of the present study propose that physical activity may benefit attentional processes. However, excessive video game play and computer use may have unfavorable influence on cognitive functions.
